# The *Radially Swollen 4* Separase Mutation of *Arabidopsis thaliana* Blocks Chromosome Disjunction and Disrupts the Radial Microtubule System in Meiocytes

**DOI:** 10.1371/journal.pone.0019459

**Published:** 2011-04-29

**Authors:** Xiaohui Yang, Kingsley A. Boateng, Li Yuan, Shuang Wu, Tobias I. Baskin, Christopher A. Makaroff

**Affiliations:** 1 Department of Chemistry and Biochemistry, Miami University, Oxford, Ohio, United States of America; 2 Biology Department, University of Massachusetts Amherst, Amherst, Massachusetts, United States of America; Tulane University Health Sciences Center, United States of America

## Abstract

The caspase-family protease, separase, is required at the onset of anaphase to cleave the cohesin complex that joins replicated sister chromatids. However, in various eukaryotes, separase has acquired additional and distinct functions. A single amino-acid substitution in separase is responsible for phenotypes of the *Arabidopsis thaliana* mutant, *radially swollen 4* (*rsw4*). This is a conditional mutant, resembling the wild type at the permissive temperature (∼20°C) and expressing mutant phenotypes at the restrictive temperature (∼30°C). Root cells in *rsw4* at the restrictive temperature undergo non-disjunction and other indications of the loss of separase function. To determine to what extent separase activity remains at 30°C, we examined the effect of the mutation on meiosis, where the effects of loss of separase activity through RNA interference are known; and in addition, we examined female gametophyte development. Here, we report that, at the restrictive temperature, replicated chromosomes in *rsw4* meiocytes typically fail to disjoin and the cohesin complex remains at centromeres after metaphase. Meiotic spindles appear normal in *rsw4* male meiocytes; however the mutation disrupts the radial microtubule system, which is replaced by asymmetric arrays. Surprisingly, female gametophyte development was relatively insensitive to loss of separase activity, through either *rsw4* or RNAi. These effects confirm that phenotypes in *rsw4* result from loss of separase activity and establish a role for separase in regulating cell polarization following male meiosis.

## Introduction

During mitosis and meiosis, sister chromatids are conjoined through sister chromatid cohesion. This cohesion holds the sister chromatids together until they are attached to the spindle and is not released until all chromosomes are properly congressed and the cell is ready for anaphase [1], [2]. Thus, sister chromatid cohesion, as well its timely release, is critical for faithful segregation of the duplicated genetic material and hence for the survival and reproduction of eukaryotic organisms. Sister chromatid cohesion is mediated in part by a complex of highly conserved proteins, referred to as the cohesin complex. The core of the cohesin complex comprises four proteins. In mitotic cells, they are *SMC1, SMC3, SCC1,* and *SCC3*, whereas in meiotic cells, *SCC1* is replaced by *REC8*.

In mitosis, cohesin is typically removed from chromosomes in two phases. First, most cohesin disassociates from chromosome arms prior to metaphase, in what is known as the prophase pathway [3], [4], [5]. This process depends in part on the phosphorylation of SCC3, which is mediated by the Polo-like kinase, Plk1, and by Wapl [6], [7], [8], [9]. In the second phase, centromeric cohesin is removed to initiate anaphase. This stepwise removal of cohesin is adaptive, insofar as linking arms throughout their length becomes less important as chromosomes condense during prophase and cohesion all along the arms impedes a synchronous anaphase onset.

At the onset of anaphase, the release of chromosome cohesion is triggered by the cysteine protease, separase, which is also known as Extra Spindle Poles 1 (ESP1) from the original mutant of yeast [10], [11], [12], [13], [14]. To release cohesion, separase specifically cleaves SCC1 [10], [15]. Prior to the onset of anaphase, the protease activity of separase is inhibited by a protein called securin. At the onset of anaphase, securin is degraded by the anaphase-promoting complex/cyclosome (APC/C).

Separase has been reasonably well studied because of its pivotal role initiating anaphase. Among the issues that have been addressed is the role of separase in meiosis, a process where the relation between chromosome arms is different from that in mitosis. Perhaps surprisingly, the cohesin pathway and the role of separase in meiosis and mitosis appear to be similar, although differences do occur.

Similar to mitotic prophase, a significant amount of cohesin is removed from the chromosome arms during meiotic prophase, in a separase-independent process, which is likely necessary for chromosome condensation [16], [17], [18], [19], [20]. At anaphase I, residual arm cohesion is removed when separase cleaves REC8, the meiotic paralog of SCC1, resulting in the resolution of chiasmata formed as a result of homologous chromosome recombination, and the subsequent separation of homologous chromosomes [12], [21], [22]. Centromeric cohesion is protected by the conserved Sgo/Mei332 family of proteins until anaphase II, when separase cleavage of REC8 facilitates the separation of sister chromatids [22], [23], [24], [25].

The role of separase in cleaving SCC1 is strongly conserved not only between mitosis and meiosis but also among eukaryotes. This pathway operates in all eukaryotes tested, including fungi, flowering plants, worms, insects, and vertebrates. Nevertheless, separases are typically large (ca. 200 kDa) polypeptides and only the protease domain, about 25% of the protein, is well conserved at the amino acid sequence level. Roles for separase, beyond cleaving cohesin, have been discovered [26]. Such roles have been elaborated most fully for budding yeast, where separase regulates microtubule dynamics in anaphase through an interaction with a regulatory phosphatase [27]. In *Caenorhabditis elegans,* separase is important for eggshell development [28], [29] apparently by regulating the incorporation of RAB-11 containing vesicles into the plasma membrane required for cytokinesis [30]. In *Drosophila melanogaster*, separase plays a role in epithelial cell re-organization and dynamics [31]; while in zebra fish, a separase mutation causes genome instability and increased susceptibility to epithelial cancer [32].

In *Arabidopsis thaliana*, separase also appears to have multiple functions. As expected, knocking down separase activity in meiocytes with RNA interference (RNAi) inhibits the removal of cohesin proteins from chromosomes during meiosis I and II, but unexpectedly also converts the symmetric radial microtubule systems that form after telophase II into asymmetric structures, partially resembling phragmoplasts [22], [33]. Such a conversion has not been reported for other meiotic mutants and implies that separase plays a role in microtubule organization or cell polarity in *A. thaliana*.

Also implicating separase in regulating microtubule function is the *radially swollen 4* (*rsw4*) mutant. This conditional mutant, isolated on the basis of temperature-dependent root swelling [34], was recently shown to harbor a mutation in separase [35]. At the restrictive temperature, cells in the *rsw4* root meristem have anomalous disjunction (typical of separase loss of function in other eukaryotes) and, in addition, have disorganized cortical microtubules and abnormally high levels of cyclin B1;1. The latter findings taken together with those on meiocytes suggest that plant separase somehow regulates microtubule function.

The mutation in *rsw4* is an alanine to valine substitution at position 603 of AtESP. This substitution might be sufficient to destabilize the enzyme against high temperature [35]. However, given that the mutation represents a relatively conservative change, and is far from the protease domain, at least some of the phenotypes seen in *rsw4* might reflect gain-of-function effects due to the presence of an aberrant protein. To gain insight into the nature of this separase mutation, we examined meiocytes of *rsw4* exposed to the restrictive temperature and compared them to the previous results with RNAi. Additionally, we took advantage of *rsw4* affecting separase in all cells and examined gametophyte development. Here we report that the *rsw4* phenotypes in meiocytes are strikingly similar to those observed with RNAi, arguing for a role for separase beyond being the traditional cleaver of cohesin.

## Results

### *The rsw4* line exhibits widespread chromosome non-disjunction in meiosis I and II

**Figure 1:**
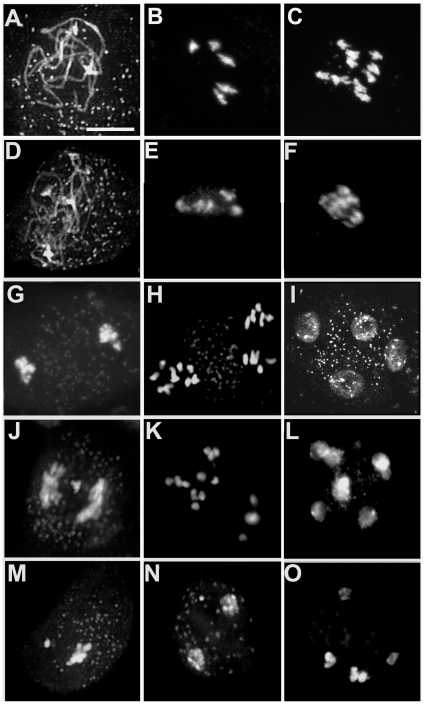
Chromosome positioning during meiosis in wild type and *rsw4*. Fluorescent micrographs of DAPI-stained chromosome spreads for wild type (A–C, G–I) and *rsw4* (D–F, J–O) exposed to 30°C for 2 days. (A, D) Pachytene. (B, E) Prometaphase I. (C, F) Early anaphase I. (G, J) Anaphase I. (H, K) Early anaphase II. (I, L) Telophase II. (M–O) Images of (M) Anaphase I. (N) Telophase I. (O) Telophase II in rsw4. Bar  =  10 µm.

The effect of the *rsw4* mutation on chromosome behavior during male meiosis was examined by analyzing chromosome spreads of meiocytes isolated from wild-type and *rsw4* plants grown at permissive (22°C) or restrictive (30°C) temperatures for two days. Growth at the restrictive temperature had no discernable effect on wild-type meiosis (Fig. 1A–C and G–I). Similarly, male meiosis in *rsw4* plants grown at 22°C was indistinguishable from that of wild-type plants (data not shown), as were meiocytes observed at stages prior to and including metaphase I in *rsw4* plants grown at 30°C (Fig. 1D, E).

Alterations in meiosis in *rsw4* plants at 30°C were first observed at the metaphase-to-anaphase I transition, when the bivalents often appeared stretched and tangled (Fig. 1F). Lagging chromosomes or chromosome fragments were observed in a number of cells at anaphase I (Fig. 1J). Chromosomal alterations were more pronounced by telophase I and metaphase II as lagging and mis-segregated chromosomes were common (Fig. 1K, M and N). Intact bivalents were observed in approximately 90% (81/92) of the cells observed at anaphase I or telophase I, suggesting that chromosome segregation was blocked by persistent cohesion.

The majority of *rsw4* meiocytes at metaphase II contained a mixture of univalents and bivalents (Fig. 1K). Many cells contained bivalents or multi-valents, dispersed in different quadrants of the cell (Fig. 1M, O). Instead of the normal tetrad of microspores observed in wild-type cells (Fig. 1I), nuclear envelopes formed around individual bivalents or groups of chromosomes/bivalents resulting in polyads with variable numbers of chromosomes (Fig. 1L). Most cells contained a mixture of univalents, bivalents and chromosome fragments, presumably giving rise to the polyads with variable numbers of nuclei.

**Figure 2:**
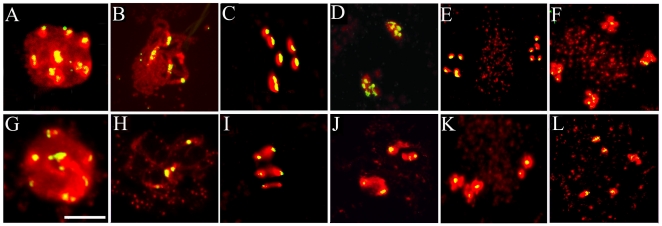
Centromere behavior during meiosis in wild type and *rsw4*. Fluorescent micrographs of chromosome spreads stained for DNA (red) and centromeres by means of in situ hybridization (yellow) of (A–F) wild type and (G–L) *rsw4* exposed to 30°C for 2 days. (A, G) Leptotene. (B, H) Pachytene. (C, I) Metaphase I to Anaphase I transition. (D, J) Telophase I. (E, K) Metaphase II. (F, L) Telophase II. Note the reduction of centromeric signal during meiosis II in *rsw4*, indicating the nondisjunction of the bivalents. Bar  =  10 µm.

To further investigate chromosomal alterations during male meiosis in *rsw4*, we examined the distribution of centromeres, using a probe that recognizes pericentromeric heterochromatin in *A. thaliana* [36]. The number of centromere signals appeared unaltered in meiocytes of wild-type plants (Fig. 2A–F) exposed to the restrictive temperature as well as in *rsw4* meiocytes (Fig. 2G–L) at both 22°C and 30°C during leptotene (Fig. 2A, G), zygotene, pachytene (Fig. 2B, H), diakinesis, and early metaphase I. The first consistent alterations in *rsw4* meiocytes were observed at the onset of anaphase I, when uneven centromere separation was observed (Fig. 2C, I). During telophase I (Fig. 2D, J) and metaphase II (Fig. 2 E, K) unevenly segregated chromosomes and bivalents resulted in fewer than ten chromosomes and centromere signals. During telophase II, bivalents were observed dispersed in the cytoplasm of *rsw4* meiocytes and significantly reduced numbers of centromeric signals were again observed (Fig. 2 L). These results demonstrate that, similar to *AtESP*-RNAi plants [22], the *rsw4* mutation blocks the disjunction of homologous chromosomes at metaphase I and sister chromatids at metaphase II. However, unlike *AtESP*-RNAi meiocytes [33], *rsw4* apparently does not cause nonspecific association of centromeres during prophase.

### Cohesin complex removal from meiotic chromosomes is defective in *rsw4*

**Figure 3:**
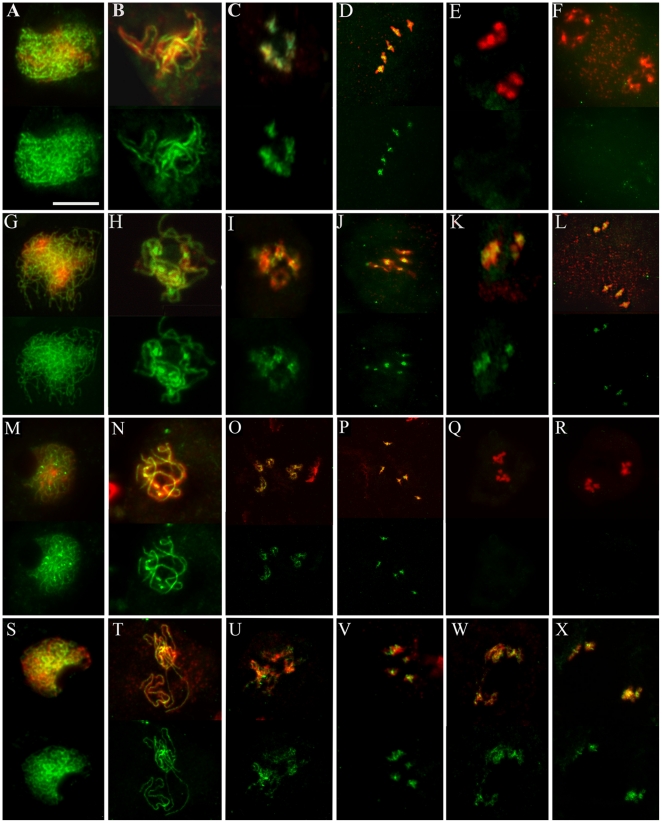
Cohesin distribution during meiosis in wild type and *rsw4*. Fluorescent micrographs of chromosome spreads stained for DNA and the meiotic cohesin subunits (A–L) SMC3 and (M–X) SYN1 in (A–F, M–R) wild type and (G–L, S–X) *rsw4* exposed to 30°C for 2 days. In each panel, the top image shows a merged image of the DNA (red) and cohesin signal (green); the bottom image only shows the cohesin signal (green) for the same meiotic spread. (A, G, M, S) Early zygotene. (B, H, N, T) Pachytene. (C, I, O, U) Diakinesis. (D, J, P, V) Metaphase I. (E, K, Q, W) Anaphase I. (F, L, R, X) Prometaphase II/Metaphase II. Bar  =  10 µm.

The meiotic alterations observed in *rsw4* plants exposed to 30°C suggest that AtESP is substantially inactive and that cohesin remains on the chromosomes. To determine whether this is so, we examined the distribution of the cohesin complex subunits, SMC3 and SYN1, in meiotic chromosome spreads. The cohesin patterns in wild type-plants exposed to 30°C were normal and resembled those previously reported for SYN1 [18]. SMC3 and SYN1 localized to the developing chromosome axes beginning at early leptotene, lined the axes of synapsed chromosomes at pachytene, were released from the chromosome arms during diplotene and diakinesis, and were barely detectable from anaphase I through metaphase II (Fig. 3A–F, M–R). Likewise, in *rsw4* exposed to 30°C, SMC3 and SYN1 patterns were normal from leptotene to metaphase I (Fig. 3G–J, S–V). However, SMC3 and SYN1 were not removed from the chromosomes and were observed into meiosis II (Fig. 3K, L, W, X). These results confirm that the meiotic non-disjunction in *rsw4* is associated with the failure to remove cohesin.

### Microtubule arrays are disrupted by the *rsw4* mutation in telophase II

**Figure 4:**
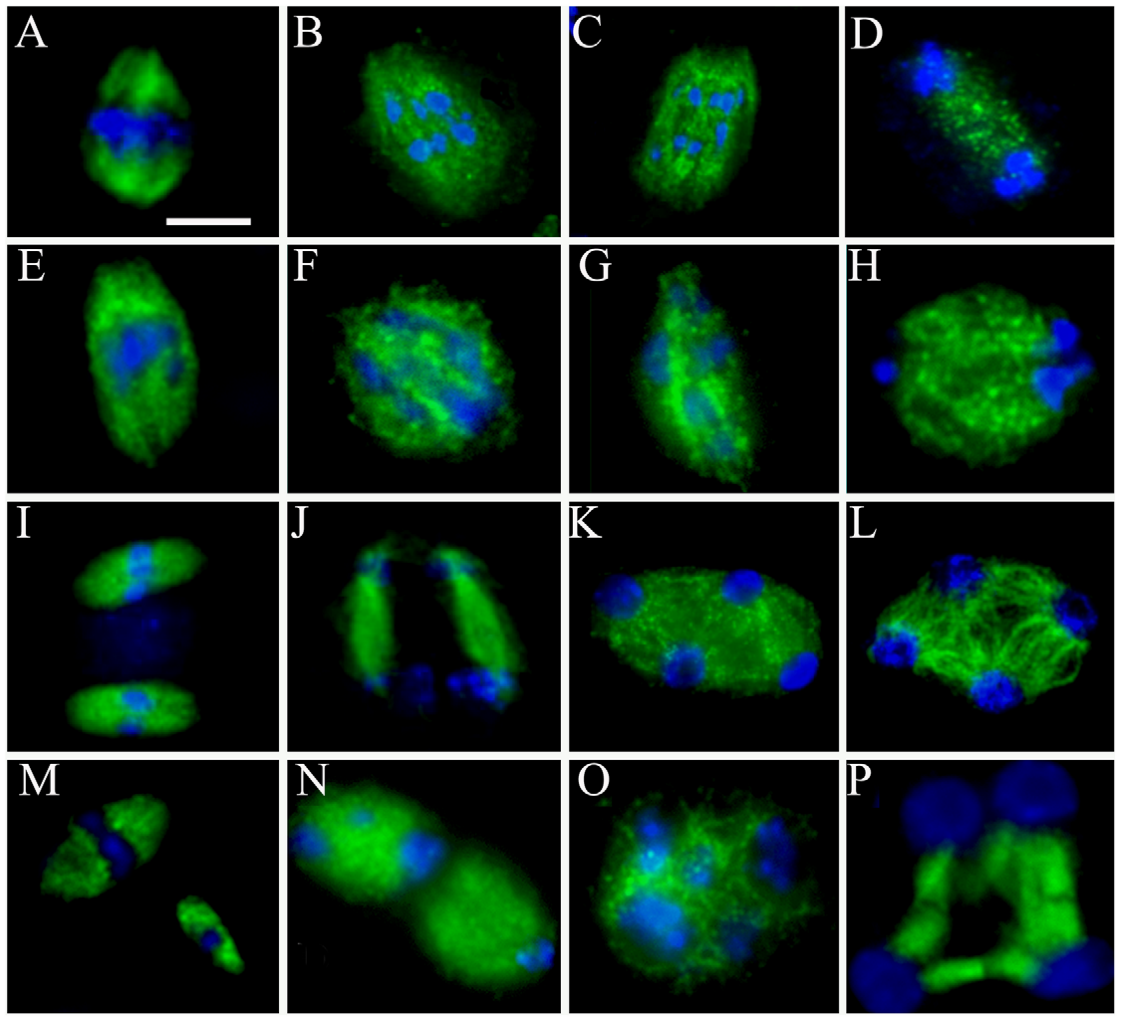
Microtubule distribution during meiosis in wild type and *rsw4*. Fluorescent micrographs of meiocytes showing microtubules (green) and DNA (blue) for (A–D, I–L) wild type and (E–H, M–P) *rsw4* exposed to 30°C for 2 days. (A, E) Metaphase I. (B, C, F, G) anaphase I. (D, H) Telophase I. (I, M) Metaphase II. (J, N) Late anaphase II. (K, O) Telophase II. (L, P) Pre-cytokenisis. Bar  =  10 µm.

In *rsw4,* the presence of numerous meiocytes with five intact bivalents at telophase II raised the possibility that reduced separase activity hinders the attachment of kinetochores to the meiotic spindle. To investigate this possibility, as well as to evaluate meiotic spindle structure, we examined microtubules. In *rsw4* exposed to 30°C, metaphase I and anaphase I spindles were bipolar (Fig. 4E–H, M–P), and generally resembled those found in wild-type plants (Fig. 4A–D, I–L). In some *rsw4* meiocytes, the spindle was broader and more diffuse than typically found in wild-type meiocytes and spindle size at metaphase II was more variable than in the wild type (Fig. 4M). Occasionally, cells had three or four spindles, with each spindle formed around chromosomes or bivalents (data not shown). All of these alterations are similar to those observed in *AtESP*-RNAi plants [33] and occur in association with abnormal chromosome disjunction; therefore we predict them to be indirect results of the loss of separase activity.

At telophase II in wild type, the meiotic spindle is replaced by a radial microtubule array that forms around each nucleus (Fig. 4K, L). In contrast, in *rsw4* exposed to 30°C, telophase II microtubule organization was unusual. In some cells, the radial organization of microtubules was weak or apparently absent (Fig. 4N, O); whereas in others, the organization was asymmetric, with prominent bundles between certain nuclear pairs and almost nothing between others (Fig. 4P). These changes to the radial microtubule system resemble the changes reported for *AtESP*-RNAi. Therefore while the loss of separase activity does not appear to affect the meiotic spindle, it does disrupt post-meiotic microtubule arrays.

### The *rsw4* mutation causes alterations in female gametophyte development

**Figure 5:**
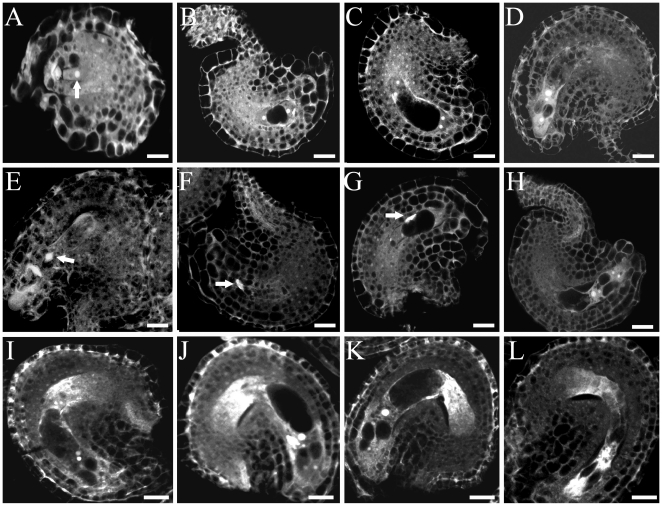
Female gametophyte development in wild type and *rsw4*. Confocal fluorescence micrographs of ovules of (A–D) wild type, (E–H) *rsw4* exposed to 30°C for 2 days, and (I –L) *AtESP-RNAi* plants. Images are the result of autofluorescence. Gametophyte development was staged according to Christensen et al. [37]. (A) FG1 embryo sac with a functional megaspore (arrow). (B) FG3 embryo sac. (C) FG 4 embryo sac. (D). FG7 embryo sac. (E) Stage FG1 in *rsw4* showing a degraded functional megaspore (arrow). (F) FG2 embryo sac showing degenerated megaspore nuclei (arrow). (G) An abnormal FG3 embryo sac with degraded nuclei (arrow). (H) A normal FG7 embryo sac. (I) FG5 embryo sac in *AtESP*-RNAi. (J) FG6 embryo sac. (K) FG7 stage embryo sac. (L) Ovule with a degraded embryo sac. Bars  =  5 µm.

The effect of the *rsw4* mutation on megagametogenesis was characterized by comparing ovules in wild type and *rsw4* plants exposed to 30°C. Female gametophyte development was staged according to Christensen et al. [37]. In wild-type plants, the functional megaspore (stage FG1) undergoes mitosis to give rise to a two-nucleate embryo sac (Fig. 5A). A central vacuole is formed between the two nuclei, separating them (stage FG3, Fig. 5B). The two nuclei then undergo a second division, giving rise to a four-nucleate embryo sac (stage FG4; Fig. 5C). After another round of division, which forms an eight-nucleate embryo sac, the two polar nuclei migrate toward the micropylar half of the developing female gametophyte (stage FG5) and fuse to form the larger central cell (stage FG6, Fig. 5D).

Abnormalities were identified in embryo sacs of *rsw4* plants as early as stage FG1. Figure 5E shows an embryo sac in which both the functional megaspore, as well as the nonfunctional meiotic products, have degenerated. Degenerated megaspores were also observed at FG2 (Fig. 5F) and FG3 (Fig. 5G). However the majority (>80%) of the embryo sacs appeared to develop normally and reached the mature FG7 stage (Fig. 5H). Therefore, although the developmental abnormalities in ovules of *rsw4* exposed to 30°C are severe, they were relatively uncommon. This is in contrast to the nearly complete penetrance for male meiotic cells and also for cells in the root tip after at least 12 h of exposure to the restrictive temperature [35]. Therefore, the *rsw4* mutation appears to have a relatively weak overall effect on female gametophyte development.

To compare the effect of inactivation of separase by the *rsw4* mutation and by RNAi, we analyzed female gametophyte development in *AtESP*-RNAi plants. Flowers from *AtESP-RNAi* plants were harvested two days after they reached stage 12C, and the pistils were fixed and subjected to confocal analysis. In wild-type plants, female gametophytes had developed to FG7 with 4 nuclei inside each ovule, almost without exception (116/118). In sliques of *AtESP*-RNAi plants, about 10% (23/172) of the ovules were at FG5 with unfused polar nuclei (Fig. 5I), approximately one half (78/172) of the ovules were at FG6 with three intact antipodal cells (Fig. 5J), while only one third (59/172) of the gametophytes were at the mature FG7 stage (Fig. 5K). Approximately 7% (12/172) of the gametophytes appeared to be degraded (Fig. 5L), which is higher than the number typically observed in wild type (1–2%), but less than the number observed in *rsw4* plants. Except for those embryo sacs that degenerated, structural abnormalities were absent from embryo sacs of *AtESP*-RNAi plants, suggesting that reduction in AtESP levels slowed or delayed the developmental program, but did not block it. Therefore, the effect of both the *rsw4* and *AtESP*-RNAi induced mutations are more pronounced on male meiosis than female gametophyte development and, further, the effects of the *rsw4* mutation appear to be somewhat distinct from those of *AtESP1*-RNAi during female gametophyte development.

## Discussion

Separase is part of an ancient mechanism designed to ensure the faithful separation of the replicated genetic material. Through its proteolytic cleavage of cohesin, separase plays a critical role in chromosome segregation that is evidently highly conserved in all eukaryotes. Nevertheless, separase has acquired additional activities in major eukaryotic taxa, making the complete functional roles of the protein in one organism difficult to predict from that of another.

In plants, separase loss of function has been studied with two approaches. In the first, male meiocytes were examined following the expression in those cells of an RNAi construct targeting separase [22], [33]. In the second, the root meristem was assayed in the temperature-dependent *rsw4* line, which harbors a point mutation in separase [35]. Such approaches are necessary because a null mutation of the separase gene is lethal, aborting the embryo after just a few divisions [22]. Both approaches found incomplete or absent chromosome disjunction as expected, but in addition reported unusual phenotypes indicative of novel separase functions. In the meiocytes, post-meiotic radial microtubule arrays were aberrant; whereas in the root, cortical microtubules were disorganized.

Here, we report that the effects on male meiocytes in *rsw4* are generally similar to those caused by *AtESP*-RNAi, including a failure to remove cohesin during metaphase, nondisjunction of chromosomes during anaphase and the appearance of altered microtubule arrays after telophase II. However, one difference between *rsw4* and *AtESP*-RNAi plants was observed. During meiotic prophase I, *AtESP*-RNAi expression prolonged the transient association among centromeres of non-homologous chromosomes [33]. This effect was not observed here in *rsw4*. Taken together these findings substantiate the canonical role of separase in removing cohesin for plant cell division and also indicate that separase regulates microtubule behavior. Although the *rsw4* mutation represents a conservative amino acid substitution that is not in the protease domain, it affects male meiosis in evidently the same way as inactivating the gene using RNA interference. These results are consistent with the hypothesis that the mutation destabilizes the protein [35]; however, whether the mutation leads to degradation of the protein (as typically happens with temperature-sensitive proteins) or only inactivation remains to be determined.

It is, however, notable that the penetrance of the *rsw4* mutation appears to vary among tissues. The mutation is highly penetrant in both roots and male meiocytes. In *rsw4* roots, abnormal anaphase figures were common after plants were exposed to 30°C for only 2 hours and were essentially ubiquitous after 12 h [35]. Likewise, almost all (81/92) *rsw4* male meiocytes had disjuction defects in anaphase I or telophase I. In contrast, less than 20% of *rsw4* female gametophytes appeared to be defective. The female gametophytes that were defective in *rsw4* appeared more or less completely aborted. This is consistent with defects in meiosis producing an aberrant female megaspore, which cannot sustain embryo sac development. That four fifths of the *rsw4* female gametophytes appeared to mature normally implies that mega-gametogenesis is substantially protected from the loss of separase function, either because the protein is stabilized or because there is a less stringent need for cohesin removal.

Conceivably, separase is protected by being synthesized before mega-sporogenesis because the *rsw4* protein is temperature-labile only (or chiefly) during its synthesis and folding. Synthesis of separase in advance of meiosis is consistent with the *AtESP*-RNAi plants also appearing to exert weak effects on mega-sporogenesis. In this line, nearly all of the female gametophytes had large embryo sacs, implying the production of reasonably functional megaspore cells. If separase were synthesized prior to female meiosis then the silencing would be ineffective from the meiosis-specific promoter (*DMC1*) that drives the RNAi construct. It is also possible that in *rsw4* the gynoecium might help protect the embryo sac from the elevated temperature, while male meiocytes might be more sensitive to the heat stress.

Nevertheless, only about a third of the *AtESP*-RNAi female gametophytes matured normally, with the rest progressing to various stages of development. At face value, this suggests that loss of separase activity dramatically slows the progression of mega-gametogenesis eventually arresting the process. However, there was no such arrest in *rsw4*. Furthermore, lines heterozygous for a T-DNA insertion in the separase gene segregate lethal embryos at 25% [22], a segregation ratio that is inconsistent with a role for separase synthesis in either male or female gametophyte linage, suggesting instead that gametophytes contain sufficient sporophytic separase to sustain their development.

Separase in plants seems to have evolved a function in regulating cell polarity. The results here confirm that loss of separase activity, whether through the *rsw4* mutation or RNAi [33], changes the appearance of the microtubule system that forms following telophase II. In wild type, microtubules from each nucleus radiate evenly into the cell, and where they encounter microtubules from another nucleus, an overlap zone forms. These overlap zones define the locus for cell plate construction and because of the symmetric growth of microtubules, this serves to partition the syncytial cell into four spores of roughly equal volume [38]. To build the cell plate, phragmoplasts do form, although the cell plates grow centripetally and contain mostly callose [39]. With loss of separase activity, microtubule arrays emanate from the nuclei asymmetrically, with dense accumulation between certain pairs of nuclei and little or no accumulation between others. In the RNAi line, cytokinesis nevertheless continues and produces spores with different volumes [33]; for *rsw4*, later states were not assayed.

A clear connection between loss of separase activity and the altered radial microtubule arrays remains to be established. Conceivably it reflects some indirect result of the disturbed disjunction, although other mutants, such as maize *dv*, that produce supernumerary micronuclei, have not been reported to disrupt the radial arrays [40]. Intriguingly, in the roots of *rsw4*, cortical microtubules are disorganized after 15 h at 30°C, and cyclinB1;1 accumulates to high levels [35]. This led the authors to hypothesize that cyclin is a target of separase activity. Although speculative, separase forms a complex with a mitotic cyclin in animal cells and cyclins accumulate in the separase mutants of both yeast and fruit flies [31], [41]. Whether or not cyclins are also misregulated in meiocytes lacking separase activity, our results support a connection between separase and the mechanisms regulating the maintenance of cell polarity.

## Materials and Methods

### Plant material

Seeds of wild-type *Arabidopsis thaliana* (Heynh) L. (Wassilewskija and Columbia), *AtESP-* RNAi, and *rsw4* were grown on commercial potting mix in a growth chamber at 22°C with a 16-h-light/8-h-dark cycle. The *AtESP*-RNAi line drives expression from the *DMC1* promoter and is described fully by Liu and Makaroff [22]. The *rsw4* line is in the Columbia background and is described in Wiedemeier et al. [35]. Flower buds with appropriate stages were collected from pre-bolting plants, fixed and analyzed as described below. For restrictive temperature treatment, plants were grown at 22°C until just before flowering when they were transferred to 30°C for two days. Subsequently buds were collected for fixation and sample preparation.

### Chromosome spreads and fluorescence in situ hybridization

The analysis of male meiosis was performed essentially as described [42] with the following modifications. Anthers were fixed in Carnoy's reagent (6∶3∶1 ethanol:chloroform:acetic acid, v/v) for 20 min and twice washed in water followed by digestion with 1.4% β-glucuronidase w/v, 0.3% cytohelicase w/v, 0.3% pectolyase w/v, and 0.3% cellulose w/v, in 10 mM sodium citrate for 40 min at 37°C. Digested anthers were washed once with 10 mM sodium citrate and cleared in 60% acetic acid for 2 min. The cells were transferred onto poly-L-lysine slides and covered with a cover slip. The slides were frozen on dry ice and the cover slips quickly removed. The dried slides were either stained with 1.5 µg/mL 4, 6-diamino-2-phenylindole dihydrochloride (DAPI) (Vector Laboratories, Inc. Burlingame, CA) or used for in situ hybridization.

To localize centromeres, fluorescence in situ hybridization was conducted on chromosome spreads prepared as above using a probe that was prepared from the 180 bp centromeric repeat sequence (CEN), which was PCR-amplified from the pAL1 clone and labeled using the Fluorescein-High Prime DNA labeling Kit (Roche, Indianapolis, IN), as previously described [43]. The CEN probe was used in hybridization solution at 5 mg/mL. The chromosomes were counterstained with DAPI and pictures were taken by using a Spot RT imaging system (Diagnostic Instruments, Inc.) attached to an Olympus fluorescence microscope (Olympus BX51).

### Antibodies and immunolocalization

SMC3, SYN1, and α-tubulin were localized in buds fixed for 2 hrs at room temperature in Buffer A containing 4% paraformaldehyde [18]. Anthers were dissected out and squashed between two perpendicular poly-L-lysine coated slides and dried overnight. Male meiocytes on the slides were covered with a thin layer of agarose/gelatin (0.94% low melting agarose, 0.84% gelatin, 0.3% w/v sucrose), then soaked in PBS for 20 min and treated with 1.4% w/v β-glucuronidase, 0.3% w/v cytohelicase, 0.3% w/v pectolyase, 0.3% w/v cellulase in 10 mM sodium citrate buffer for 30 min at 37°C. After washing in PBS the slides were blocked in buffer (PBS, 5% w/v BSA) for 60 min and incubated overnight at 4°C in a moist chamber with primary antibody (1∶300). The rabbit polyclonal antiserum against SYN1 and SMC3 have been described [18], [44]. Mouse anti-tubulin antibody was obtained from the Developmental Studies Hybridoma Bank. Slides were washed eight times (20 min each) with buffer (PBS, 1 mM EDTA, 0.1% of Tween 20) and the primary antibody detected with Alexa Fluor 488 goat anti-rabbit secondary antibody (1∶500) (Molecular Probes) with or without Alexa Fluor 594 goat anti-mouse secondary antibody (1∶500) overnight at 4°C, washed again as above and mounted.

Samples were viewed with an Olympus 1X81 fluorescence deconvolution microscope system. Data were analyzed with Image Pro Plus (Media Cybernetics; http://www.mediacy.com) and organized with Photoshop (Adobe System, San Jose, CA, USA). Meiotic stages were assigned based on chromosome and cellular morphology as well as the stage of the surrounding tissue.

### Analysis of female gametophyte development

A confocal fluorescence microscope was used to analyze ovule development in *rsw4* plants, based on methods described previously [37]. Briefly, inflorescences were collected and fixed in 4% glutaraldehyde under vacuum overnight, dehydrated in a graded ethanol series (20% steps for 1 hr each) and cleared in a 2∶1 mixture of benzyl benzoate:benzyl alcohol. The pistils were mounted under sealed coverslips. Autofluorescence associated with the samples were visualized and images collected with Olympus Flouview 2.0 software (http://www.olympus-global.com/) and analyzed with Image Pro Plus.
